# Multimodal Anion-Exchange Resins for Chromatographic Separation of Antibody Aggregates

**DOI:** 10.3390/biom16060785

**Published:** 2026-05-27

**Authors:** Simona Kotuličová, Tomáš Molnár, Milan Polakovič

**Affiliations:** Department of Chemical and Biochemical Engineering, Institute of Chemical and Environmental Engineering, Faculty of Chemical and Food Technology, Slovak University of Technology, Radlinského 9, 812 37 Bratislava, Slovakia; simona.kotulicova@stuba.sk (S.K.); tomas.molnar1@stuba.sk (T.M.)

**Keywords:** antibody aggregates, multimodal chromatography, agarose matrix, BMEA ligand, pore accessibility, monomer/aggregate separation

## Abstract

Efficient protein aggregate removal remains a major challenge in downstream bioprocessing because high aggregate clearance must be achieved without compromising monomer yield. Mixed-mode chromatography (MMC) has emerged as a promising approach, offering enhanced selectivity through combined ionic and hydrophobic interactions and salt-tolerant behavior. However, the relative roles of matrix pore accessibility and ligand density remain insufficiently understood. In this study, MMC adsorbents based on 4% and 6% agarose matrices were functionalized with a BMEA ligand. Inverse size-exclusion chromatography revealed that functionalization caused matrix syneresis, increasing dry matter content to 23% and enhancing mechanical rigidity. MMC-Ag4, with a larger mean pore radius (19.1 nm), exhibited a selectivity factor of 2 toward aggregates in static binding experiments, whereas the denser MMC-Ag6 (15.7 nm) showed no selectivity. In column studies using a feed containing 10% aggregates, MMC-Ag4 outperformed the commercial benchmark Capto Adhere, achieving monomer yields of 80–90% at 97–98% purity with salt tolerance up to 300 mM NaCl. These findings indicate that while MMC-Ag6 is limited by pore blockage, the optimized pore accessibility of MMC-Ag4 enables effective aggregate recognition. In conclusion, multimodal adsorbent design must balance ligand density with matrix porosity to ensure high resolution and yield in aggregate removal.

## 1. Introduction

The integration of therapeutic proteins into drug development pipelines is growing rapidly [1,2]. However, the development of robust, commercial-scale purification processes for recombinant protein therapeutics remains a major challenge, particularly due to the propensity for irreversible protein aggregation during various stages of the manufacturing process [2,3,4]. Protein aggregation, characterized by the abnormal association of partially unfolded or misfolded proteins into oligomeric and higher-order structures, is a common phenomenon in protein drug development [5,6,7,8]. Given the strong correlation between protein aggregation and numerous human diseases, the presence of aggregates in therapeutic formulations is considered a critical quality concern that may compromise regulatory approval [4,5,9].

Consequently, substantial effort is devoted to aggregate removal during downstream processing, which can account for more than 60% of total manufacturing costs [10,11]. Chromatography plays a pivotal role in addressing these quality challenges and serves as a cornerstone of purification strategies to ensure product safety and efficacy [12,13,14,15]. Conventional downstream processing for monoclonal antibodies typically comprises a Protein A capture step, followed by two polishing chromatography steps. These polishing steps commonly employ cation and anion exchange chromatography (CEX/AEX) to reduce impurities, including host cell DNA, host cell proteins (HCP), aggregates, leached Protein A, and potential viral contaminants [13,16,17]. Hydrophobic interaction chromatography (HIC) is also widely used in polishing workflows [17,18,19], particularly for the removal of protein aggregates, which often exhibit increased surface hydrophobicity [14].

Mixed-mode chromatography, also referred to as multimodal chromatography (MMC), represents a promising cost-effective alternative in pharmaceutical and biopharmaceutical applications [13,14,20,21,22]. MMC relies on multiple interaction mechanisms between the stationary phase and solutes [20,23,24,25,26]. This feature enables its use in diverse applications, including capture steps for therapeutics such as monoclonal antibodies (mAbs) [16,27], vascular endothelial growth factor (VEGF) [28,29], and deoxyribonucleic acid (DNA) [30]. Unlike conventional single-mode resins, the efficiency and selectivity of protein adsorption on mixed-mode resins can be tuned by adjusting process parameters such as ionic strength and pH, thereby modulating hydrophobic and electrostatic interactions between the protein and the ligand [24,31,32].

In antibody purification, the integration of multimodal chromatography as a post-Protein A polishing step may reduce the number of chromatography steps and enhance overall process productivity [13,15]. For this purpose, MMC offers improved selectivity, salt-tolerant binding, and high loading capacity [15]. Zhang et al. [33] demonstrated that the multimodal cation-exchange resin Capto MMC ImpRes effectively removes aggregates in the bind-elute mode with linear gradient elution. Furthermore, multimodal anion-exchange resins such as Capto Adhere, PPA Hypercel, HEA Hypercel, and MEP Hypercel are widely applied for aggregate removal [14].

Pezzini et al. [34] showed that these resins can also efficiently capture antibodies and significantly reduce HCP levels during the capture step. In addition, Capto Adhere has been reported to outperform traditional AEX resins in aggregate removal and to exhibit a distinct binding profile toward CHO-derived HCPs [35]. In efforts to develop cost-effective alternatives of Protein A chromatography, Maria et al. [36] proposed a purification platform employing two MMC resins and an AEX membrane. Similarly, Toueille et al. [37] demonstrated that two carefully optimized MMC steps could replace Protein A-based capture entirely.

In this study, novel MMC resins based on agarose-based matrices with different polymer concentration were functionalized with N-benzyl-N-methylethanolamine (BMEA) ligand to examine the impact of matrix pore size and ligand density for effective protein aggregate removal. The synthesis of MMC resins in the present study adheres to a well-established synthetic route involving the activation of the resins with allyl bromide [38] and subsequent bromination with N-bromosuccinimide [39], a method that was pioneered by Burton and Harding to achieve high-density ligand attachment. This strategy was subsequently demonstrated by Gao et al. in the preparation of a benzylamine functionalized matrix, which exhibited robust salt-tolerant binding properties [40].

A subsequent study of Gao et al. showed that in-house prepared resins synthesized via the allyl bromide/N-bromosuccinimide activation route achieved antibody monomer/aggregate separation performance comparable to that of commercial benchmarks such as Capto Adhere [14]. Furthermore, the investigation of different agarose matrices is justified by evidence that pore size and ligand density significantly influence the mass transport and adsorption capacity of large biomolecules such as IgG [41,42,43].

Building on these foundations, the present study evaluates the effect of agarose matrix composition on the performance of MMC resins in the separation of IgG monomers from high-molecular-weight aggregates. The separation performance of the individual resins was compared with that of commercial multimodal anion-exchange resin Capto Adhere, with particular emphasis on product purity, antibody monomer yield, and process productivity.

## 2. Materials and Methods

### 2.1. Materials

Non-functionalized agarose gels 4% B Agarose Bead and 6% B Agarose Bead from Agarose Bead Technologies (Madrid, Spain) were used for the preparation of multimodal adsorbents. Capto Adhere was purchased from Cytiva (Uppsala, Sweden). The ligand BMEA (90% purity) was obtained from Sigma-Aldrich (St. Louis, MO, USA). The polyclonal antibody preparation Gammanorm (total protein concentration 165 mg/mL) was kindly provided by Octapharma (Vienna, Austria). Salts and other analytical grade chemicals were supplied by Mikrochem (Pezinok, Slovakia). Glucose (Sigma-Aldrich, St. Louis, MO, USA) and dextrans (Fluka BioChemika, Buchs, Switzerland, and Sigma-Aldrich, St. Louis, MO, USA) with weight-average molecular weights 1200, 6000, 9300, 17,500, 56,000, 70,000, 110,000, 220,000, and 2,000,000 were used as solute probes.

### 2.2. Feed Preparation and Characterization

The Gammanorm preparation was diluted with 10 mM acetate buffer (pH 5.0) to an antibody concentration of 16.5 mg/mL. Aggregation in this solution was induced by thermal stress at 60 °C for 48 h following a protocol described by Molnár et al. [44]. The concentrations of IgG monomers and total aggregates were determined by size-exclusion chromatography [44]. Changes in the mean hydrodynamic radius of the protein species over time were monitored by dynamic laser light instrument using a Zetasizer Ultra Red with ZS XPLOTER 3.30.42 software (Malvern Panalytical, Malvern, UK). Based on six replicate measurements, the initial Gammanorm preparation contained approximately 96% IgG monomers (mean hydrodynamic radius, *r*_h_ ≈ 5–5.5 nm) and 4% aggregates, predominantly dimers (*r*_h_ ≈ 7–8 nm). After 48 h incubation at 60 °C, the mass fractions of IgG monomers and aggregates were 58% and 42%, respectively, with approximately 90% of the aggregates consisting of dimers and trimers (*r*_h_ ≈ 9–10 nm). The resulting monomer/aggregate mixture was combined with a 96% monomer solution in a citrate buffer (pH 6.0) containing NaCl concentrations ranging from 0 mM to 300 mM. The aggregate content was adjusted to 10% at a total antibody concentration of 16.5 mg/mL.

### 2.3. Resin Preparation and Characterization

#### 2.3.1. Resin Preparation

As an initial step in resin preparation, allylation of the agarose gel was performed in parallel in multiple vials. To activate the agarose matrix, 2.7 g ± 0.1 g of agarose gel was suspended in 17.63 mL ± 0.65 mL of 2 M NaOH and incubated for 5 min. The required volume of NaOH was calculated based on the amount and molar mass of agarose. Subsequently, 3.05 mL ± 0.11 mL of allyl bromide was added to the reaction mixture to promote the substitution reaction. The suspension was incubated for 24 h at 25 °C on a rotary shaker (Multi RS-60, BioSan, Riga, Latvia).

The particles bearing the spacer arm were washed thoroughly three times with redistilled water (15 min per step), followed by washing with isopropyl alcohol (10 min) and final rinse with redistilled water. The spacer arm was subsequently activated by bromination. First, 9.36 mL ± 0.35 mL of 0.6 M sodium acetate solution was added. The particles were then temporarily removed from the suspension, and bromine water was added until the solution turned deep orange. The particles were then returned to the solution, and the suspension was incubated under continuous agitation on the rotary shaker (10 rpm) for 15 min.

The residual bromine was quenched with 0.64 mL ± 0.02 mL of 5 wt% sodium thiosulfate pentahydrate (Na_2_S_2_O_3_·5H_2_O) solution. The activated particles were washed with redistilled water and twice with 20% (*v*/*v*) ethanol. They were then transferred directly into 30.2 mL ± 1.12 mL of BMEA reaction solution prepared by dissolving BMEA (60 wt%) in 20% ethanol and adjusting the pH to 12 with 0.1 M NaOH. The mixture was incubated for 20 h at 50 °C on the rotary shaker. The prepared resins were thoroughly washed with 20% ethanol and redistilled water before use.

For the purpose of this study, the prepared MMC resins bearing the BMEA ligand and based on the 4 and 6% agarose matrices are hereafter referred to as MMC-Ag4 and MMC-Ag6.

#### 2.3.2. Resin Characterization

The dry mass fraction of the resins, *w*, was determined gravimetrically. A defined mass of the resin was weighed and subsequently dried in a vacuum dryer at 60 °C until constant weight was achieved. The dry mass fraction was used to calculate the specific pore volume (per mass of dry resin), *v*_p_, which is a suitable measure for comparing adsorbents used in the separation of monoclonal antibodies [45].

The total ligand density of the functionalized resins was determined by acid-based-titration using an ÄKTA avant 150 system (Cytiva, Uppsala, Sweden). The resin particles were packed into a 1 mL Tricorn 5/50 column (Cytiva, Uppsala, Sweden). Initially, the stationary phase was equilibrated with 0.1 M NaOH to ensure that the positive charge of the BMEA ligands was saturated with hydroxide ions. The bed was then rinsed with water to remove any unbound ions. The titration process involved applying 10 mM HCl to the column while measuring the conductivity of the permeate in-line. After neutralisation of the hydroxide ions, an increase in conductivity was observed, consequent to the presence of excess hydrogen ions. The ligand density (Λ) was calculated from Equation (1):

(1) $$\Lambda =\frac{((V_{HCl}-V_{tot})\cdot \Lambda_{max}-A_{evP})\cdot \frac{10}{\Lambda_{set}}}{m_{M}}$$

where $V_{HCl}$ is the volume of HCl solution (mL), $V_{tot}$ is the dead volume of the chromatography system (mL), $\Lambda_{max}$ is the experimentally measured conductivity of the 10 mM HCl solution (mS/cm), $\Lambda_{set}$ is the theoretical conductivity of 10 mM HCl at 25 °C used for signal normalization (mS/cm), $A_{evP}$ is the integrated conductivity peak area corresponding to excess hydrogen ions (mL·mS/cm), and $m_{M}$ is the resin mass (g).

The pore structure of the resins was examined by inverse size-exclusion chromatography (ISEC), in accordance with established protocols [45,46]. Chromatographic experiments were performed in a Tricorn 5/50 column packed with the resin particles. The mobile phase consisted of a 20 mM phosphate buffer containing 100 mM NaCl at pH 7. Solute probes of different molecular sizes, including glucose and dextran polymers with molecular weights ranging from 1200 to 2,000,000 g/mol, were injected at a concentration of 5 g/L.

The column effluent was monitored using the refractive index detector of an Agilent Technologies 1100 HPLC system (Santa Clara, CA, USA). Solute retention times ($t_{R}$) were determined from the first absolute moment of the residence time distribution. The distribution coefficient ($K_{p}$) was calculated using following relationship:

(2) $$K_{p}=\frac{t_{i}-t_{max}}{t_{min}-t_{max}}$$

where $t_{i}$ is the retention time of the injected solute probe, and $t_{min}$ and $t_{max}$ are the retention times of the glucose and the largest dextran, respectively.

The hydrodynamic radius ($r_{h}$) of each probe was estimated using the Mark-Houwink–Sakurada relationship:

(3) $$r_{h}=0.027M_{w}^{0.5}$$

where $M_{w}$ is the weight-average molecular weight of the solute.

### 2.4. Batch Adsorption Experiments

Batch adsorption experiments were conducted at pH 6 with NaCl concentrations ranging from 0 mM to 300 mM. Prior to use, the resin particles were washed with redistilled water and equilibrated with 20 mM citrate buffer. Approximately 50 mg of resin was transferred into a cartridge with a microfilter (Macherey-Nagel Chromabond, Düren, Germany) and 1 mL of feed solution at the desired NaCl was added. Adsorption was carried out at room temperature on a rotary shaker for 1 h; preliminary kinetic experiments confirmed that this contact time was sufficient to reach equilibrium.

The bulk liquid equilibrium concentrations of total protein and aggregates ($c_{a}$) were determined. The equilibrium concentration of IgG monomer $(c_{m}$) was obtained as the difference between the total protein concentration and the aggregate concentration [44]. The equilibrium adsorbed amounts of IgG aggregates and monomers per mass of wet resin ($q_{a}$ and $q_{m}$, respectively) were calculated from mass balances. To quantify preferential adsorption of aggregates over monomers, the selectivity coefficient S was calculated as:

(4) $$S=\frac{q_{a}/c_{a}}{q_{m}/c_{m}}$$

All experiments were performed in duplicate. For reuse, the resins were washed with redistilled water, bound proteins were eluted with 20 mM citrate buffer (pH 4.5) for 1 h, and the resins were subsequently regenerated with 1 M NaOH.

### 2.5. Chromatographic Experiments

Fixed-bed separation experiments were performed using MMC-Ag4, MMC-Ag6, and Capto Adhere resins, packed into Tricorn 5/50 (Cytiva, Uppsala, Sweden) chromatographic columns connected to an ÄKTA FPLC system (Cytiva, Uppsala, Sweden). The compositions of feed, eluent and regeneration solutions were identical to those used in the batch adsorption experiments described in Section 2.4.

In the first step, the chromatographic bed was equilibrated with 20 column volumes (CV) of adsorption buffer. Next, 1 mL of feed solution was loaded onto the column. Unbound proteins were washed out with adsorption buffer. Elution was performed using 10 CV of 20 mM citrate buffer (pH 4.5). During elution, 1 mL fractions were collected, and monomer and aggregate concentrations were determined. Corresponding yields and purities were calculated for the pooled fractions. The adsorbents were regenerated with 0.5 M NaOH solution.

### 2.6. Antibody Sample Analyses

Protein concentrations in the samples were determined by ultraviolet (UV) absorbance at 280 nm and by a bicinchoninic acid (BCA) assay [47], using bovine serum albumin (BSA) and Gammanorm (Immunoglobulin G) as reference standards. Aggregate content was determined using a Nile Red fluorescence assay [44].

## 3. Results and Discussion

### 3.1. Resin Matrix Hydration and Textural Properties

In this study, MMC-Ag4 and MMC-Ag6 resins were prepared to evaluate their efficiency in separating antibody monomers from protein aggregates. In addition to adsorption performance, several physicochemical properties of the prepared adsorbents were characterized. Table 1 summarizes the measured dry mass contents of these multimodal chromatography resins; values for the native gel beads are included to illustrate the effect of activation and functionalization. Only small differences were observed between resins based on 4% and 6% agarose. However, the functionalization caused a pronounced increase in dry matter content, from approximately 5–6% in the native resins to 22–23% in the modified materials. This substantial increase cannot be attributed solely to the attachment of the allyl spacer arm and BMEA ligand, but instead indicates significant syneresis of the gel matrix.

This effect is further reflected in the calculated values of specific pore volume per mass of dry resin, a commonly used parameter for assessing chromatographic adsorbents. The native 4% and 6% agarose beads exhibited values of 18.5 mL/g and 14.6 mL/g, respectively, which are typical soft agarose gels used in size-exclusion chromatography. In contrast, MMC-Ag4 and MMC-Ag6 showed markedly lower specific pore volumes of 3.3 mL/g and 3.4 mL/g, respectively, consistent with more rigid gel structures [46,48]. The preparation procedure therefore substantially improved the mechanical rigidity of the resulting MMC resins. Further studies using dedicated structural characterization methods would be required to determine whether this extent of shrinkage involves irreversible changes in the pore structure and whether it can be moderated by modified activation or functionalization conditions.

### 3.2. Resin Pore Accessibility and Size Distribution

The pore accessibility of both adsorbents was examined by inverse size-exclusion chromatography using glucose and dextran polymers as molecular probes. Only very small differences were observed in the total pore volume of these two resins determined as the difference between the retention volumes of the smallest and largest probes. The total pore volumes measured by ISEC differed by only 1%, consistent with the dry weight measurements.

**Figure 1:**
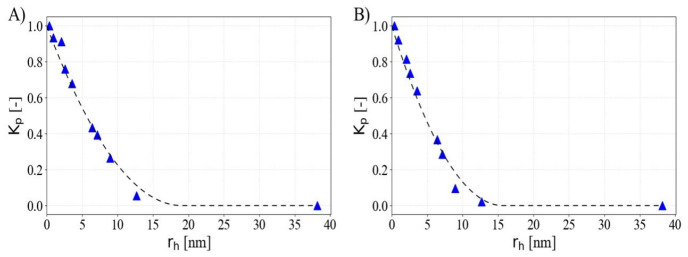
Pore accessibility of multimodal adsorbents (**A**) MMC-Ag4 and (**B**) MMC-Ag6 determined by inverse size-exclusion chromatography. Curves represent fits of the experimental *K_p_*–*r_h_* dependence using the cylindrical pore model (Equation (5)).

However, Figure 1 shows that the pore structures of MMC-Ag4 (Figure 1A) and MMC-Ag6 (Figure 1B) are similar but not identical. The dependence of the pore distribution coefficient, *K*_p_, on the hydrodynamic radius, *r*_h_, of dextran probes is nearly identical for both resins at *r*_h_ ≤ 5–6 nm. *K*_p_ decreases approximately linearly from 1 to about 0.5 at this threshold value of *r*_h_. Above this threshold, the *K*_p_ dependences for MMC-Ag4 and MMC-Ag6 diverge. *K*_p_ for MMC-Ag6 decreases more rapidly and reaches the exclusion limit at *r*_h_ ≈ 15 nm, whereas the exclusion limit for MMC-Ag4 occurs at *r*_h_ ≈ 20 nm. This implies a larger mean pore size for MMC-Ag4.

To estimate the mean pore size of both resins, the cylindrical pore model (Equation (5)) was employed to fit the data presented in Figure 1 [45]. Besides the approximation of pore texture by cylindrical geometry, another significant assumption of this model formulation is a monodisperse pore size distribution. The model contains a single parameter, the pore radius, *r*_p_:

(5a) $$K_{p}={\left(1-\frac{r_{h}}{r_{p}}\right)}^{2}\;\;\;r_{h}<r_{p}$$

(5b) $$K_{p}=0\;\;\;\;\;\;\;r_{h}\geq r_{p}$$

This simple model provided an excellent fit to the *K_p_*–*r_h_* dependence of both resins, as shown in Figure 1. The standard deviations were 4.7% and 4.6% for MMC-Ag4 and MMC-Ag6, respectively, with no systematic bias observed. The fitted values of *r*_p_ were 19.1 nm for MMC-Ag4 and 15.7 nm for MMC-Ag6. In general, ISEC data are limited in resolution and do not permit determination of the complete pore size distribution [45]. Nevertheless, the close agreement with the monodisperse pore-size distribution model suggests that both resins possess a relatively narrow pore size distribution [45,48].

The characteristic pores of these resins can be classified as large mesopores according to IUPAC recommendations [49]. It must be noted that these recommendations specify the upper mesopore boundary by the pore width, i.e., pore diameter, at 50 nm. Thus, the pore diameter of 30 nm for MMC-Ag6, and especially 40 nm for MMC-Ag4, are close to the threshold between mesopores and macropores. In addition, it can be assumed that the fraction of smaller mesopores or micropores has a very limited contribution to the matrix porosity and negligible impact on the accessibility of antibody molecules to internal binding sites.

These results indicate that the structural integrity and hydration of the agarose matrix were maintained during activation and coupling. No excessive matrix contraction or pore blockage occurred that could have a detrimental effect on the antibody pore accessibility. However, the mean pore sizes of the resins allow a cautious quantitative analysis of this accessibility. When applying the cylindrical pore model to IgG monomers and aggregates, it must be noted that these molecules are not spherical, which may introduce some bias into the calculated distribution coefficients.

Considering the IgG monomer radius (*r*_h_ ≈ 5–5.5 nm), the *K_p_* of the resins will be around 0.5, with the value for MMC-Ag4 being about 15% higher. However, for an aggregate with *r*_h_ = 10 nm, the model predicts *K_p_* values of 0.23 for MMC-Ag4 and only 0.13 for MMC-Ag6. These lower *K_p_*-values do not necessarily imply lower binding capacities of IgG dimers and trimers compared to monomers. They indicate that intraparticle liquid concentrations will be proportionally lower, though potentially still high enough to saturate the ligands. Nevertheless, a simple geometrical consideration of a 10 nm molecule bound to the wall of a 15.7 nm pore suggests that such a pore would be effectively blocked for other molecules of this size. In conclusion, the 20–25% difference in pore size between MMC-Ag4 and MMC-Ag6 may result in resin selectivity toward aggregate binding.

### 3.3. Surface Functionalization and Adsorption Characteristics

Ligand immobilization is a key determinant of multimodal adsorbent performance, as it governs binding capacity, selectivity, and the balance of interaction modes involved in protein retention. Therefore, the density of functional ligands introduced onto the agarose matrix was quantified and related to the adsorption characteristics of the prepared resins.

The ligand densities (per wet resin mass), determined by titration of ionized functional groups, were 77 μmol/g for MMC-Ag4 and 47 μmol/g for MMC-Ag6. Although these values are lower than those typical of ion exchange resins with a grafted layer used in antibody separations [50,51], they are appropriate for multimodal adsorbents, where moderate ligand densities help maintain accessibility and balanced interaction modes. Notably, MMC-Ag6 exhibited a lower ligand density despite its denser matrix structure, suggesting that steric constraints within the tighter gel network may limit the extent of ligand immobilization.

Table 2 summarizes the results of batch adsorption experiments performed with an IgG monomer/aggregate mixture at a total protein concentration of 16.5 mg/mL containing 10% aggregates. In preliminary experiments, the pH was varied from 5 to 8; however, pH 6.0 was ultimately selected for both batch and column studies. At this pH, the IgG surface carries only a limited number of negatively charged groups [52], and for the feed containing 10%, hydrophobic interactions with the BMEA ligand are therefore expected to be more pronounced. Adsorption experiments were conducted at four different NaCl concentrations from 0 mM to 300 mM in duplicate. As no significant effect of salt concentration was observed, the equilibrium values reported in Table 2 represent the mean and standard deviation calculated from eight measurements.

Both resins exhibited pronounced multimodal behaviour and strong salt tolerance. Notably, the total amount of adsorbed protein was similar for both adsorbents despite the lower ligand density of MMC-Ag6. However, MMC-Ag6 bound antibody aggregates slightly less strongly than MMC-Ag4, resulting in a substantial difference in selectivity. Binding on MMC-Ag6 was essentially nonselective, whereas MMC-Ag4 showed preferential binding of aggregates, with a selectivity factor close to 2.

These results suggest that, although both materials provide comparable binding capacity, the broader pore structure and higher ligand accessibility of MMC-Ag4 enhance aggregate recognition and retention. Consequently, MMC-Ag4 appears to be the more promising candidate for dynamic separation experiments aimed at selective aggregate removal. The observed salt tolerance and moderate binding strength at pH 6 further indicate suitable operating conditions for subsequent column studies.

### 3.4. Chromatographic Separation of IgG Monomers and Aggregates

Following the results of the batch adsorption experiments, elution chromatography experiments were designed to further examine the separation performance of the multimodal resins. The primary focus was on MMC-Ag4, although selected experiments were also conducted with MMC-Ag6. In all experiments, the feed consisted of an IgG monomer/aggregate mixture; as in the batch studies, the total protein concentration was 16.5 mg/mL with an aggregate content of 10%.

**Figure 2:**
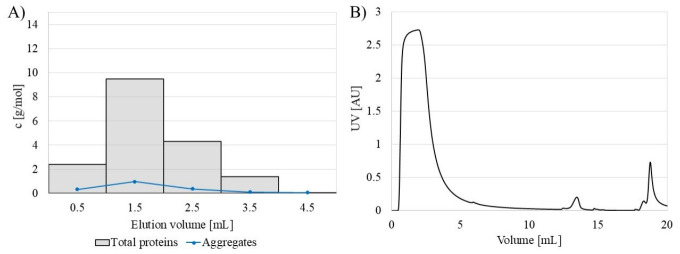
Elution and regeneration phases of chromatographic separation of IgG monomers and aggregates using the multimodal adsorbent MMC-Ag6 with a feed containing 300 mM NaCl. (**A**) Compositions of collected fractions: total protein (grey bars) and aggregates (blue circles). (**B**) UV absorbance at 280 nm.

Chromatographic experiments for the MMC-Ag6 resin, for which twelve separation experiments were conducted at four different NaCl concentrations, provided separation behaviour consistent with the equilibrium characteristics determined in batch experiments. Figure 2 shows a representative chromatogram for this resin. Consistent with the batch studies, no effect of salt concentration and no selectivity between monomer and aggregate binding were observed. Each collected fraction contained approximately 10% aggregates, identical to the feed composition (Figure 2A). The UV absorbance profile in Figure 2B indicates relatively weak binding of both species, as they were almost completely eluted with the elution buffer, while only a small fraction remained bound and was removed during the regeneration step.

**Figure 3:**
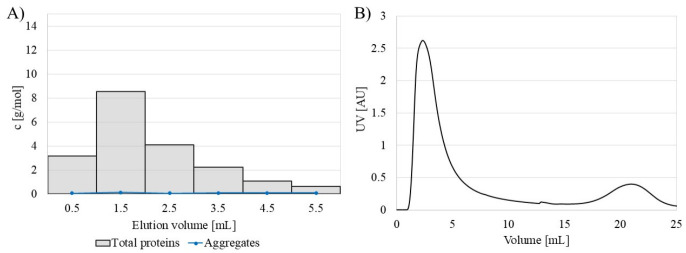
Chromatographic separation of IgG monomers and aggregates on MMC-Ag4 with a feed containing no NaCl. (**A**) Compositions of collected fractions: total protein (grey bars) and aggregates (blue circles). (**B**) UV absorbance at 280 nm. A monomer yield of 70% was achieved at 98% purity, while 80% yield corresponded to 97% purity.

**Figure 4:**
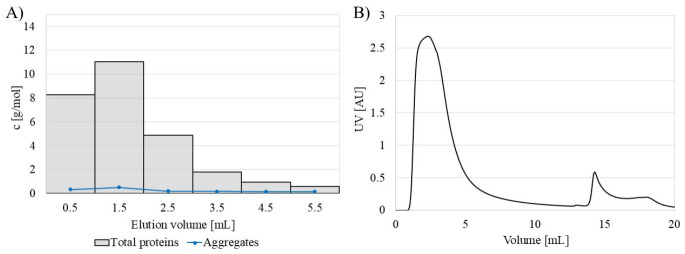
Chromatographic separation of IgG monomers and aggregates on MMC-Ag4 with a feed containing 50 mM NaCl. (**A**) Compositions of collected fractions: total protein (grey bars) and aggregates (blue circles). (**B**) UV absorbance at 280 nm. A monomer yield of 80% was achieved at 96% purity, while 90% yield corresponded to 95% purity.

**Figure 5:**
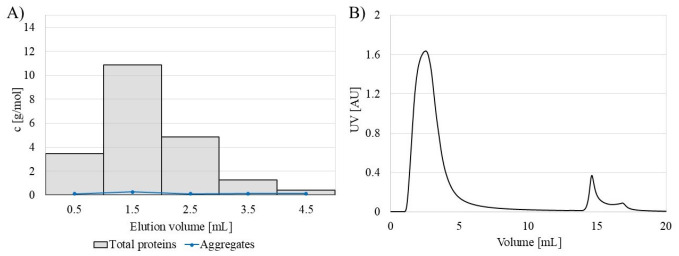
Chromatographic separation of IgG monomers and aggregates on MMC-Ag4 with a feed containing 150 mM NaCl. (**A**) Compositions of collected fractions: total protein (grey bars) and aggregates (blue circles). (**B**) UV absorbance at 280 nm. A monomer yield of 85% was achieved at 97% purity, while 90% yield corresponded to 95% purity.

**Figure 6:**
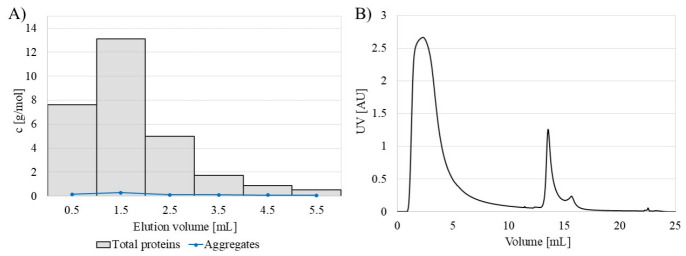
Chromatographic separation of IgG monomers and aggregates on MMC-Ag4 with a feed containing 300 mM NaCl. (**A**) Compositions of collected fractions: total protein (grey bars) and aggregates (blue circles). (**B**) UV absorbance at 280 nm. A monomer yield of 90% was achieved at 98% purity.

Ten separation experiments were conducted for MMC-Ag4 at the same four NaCl concentrations. Consequently, all separations were performed at least in duplicate. Figure 3, Figure 4, Figure 5 and Figure 6 present selected chromatograms for each salt concentration. Plots A show the compositions of the fractions collected during the elution phase and, while plots B display the UV absorbance at 280 nm for both the elution and regeneration phases. The latter indicate that over 90% of the proteins were eluted during the elution phase, with less than 10% retained and subsequently recovered during the regeneration phase.

Plots A demonstrates a very low outlet aggregate concentration. Except for a few collected fractions, the values can barely be distinguished from zero. The breakthrough stream thus provides a separated product—high purity monomer. The legends of Figure 3, Figure 4, Figure 5 and Figure 6 specify the purities and corresponding yields in the first 3–4 pooled fractions. A typical purity value was 97–98%, with monomer yields ranging from 80% to 90%. Since the fractions are relatively large, the yields are rounded to tens of percent. Nevertheless, the yields are high considering the achieved purity.

The purity is also higher than that inferred from the selectivity obtained in the batch equilibrium experiments. However, this selectivity pertains only to the loading phase of the chromatographic experiments. It is possible that elution at pH 4, which favors hydrophobic interactions, caused selective desorption of monomers, thereby enhancing the purification effect. Furthermore, the peak monomer concentrations and yield values suggest that the purification effect may have been further intensified with increasing NaCl concentration.

**Figure 7:**
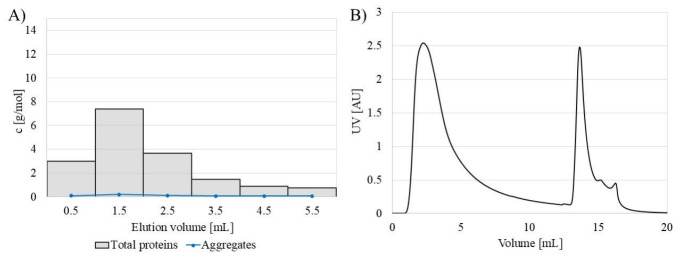
Chromatographic separation of IgG monomers and aggregates on Capto Adhere with a feed containing 150 mM NaCl. (**A**) Compositions of collected fractions: total protein (grey bars) and aggregates (blue circles). (**B**) UV absorbance at 280 nm. A monomer yield of only 60% was achieved at 97% purity.

As previously mentioned, Capto Adhere is a widely used benchmark multimodal anion exchanger for monoclonal antibody purification. Consequently, a similar set of chromatographic experiments was conducted as for MMC-Ag4 and MMC-Ag6. Since no pronounced effect of NaCl concentration was observed, only one illustrative chromatogram is shown in Figure 7. Comparing this experiment with those presented in Figure 3, Figure 4, Figure 5 and Figure 6 reveals two distinct features.

Firstly, the monomer fraction peaks were flatter, with reduced peak maxima. Secondly, monomer yields were lower, and a significantly larger fraction of the total protein was retained during the elution phase, with approximately one third being recovered only during resin regeneration. Thus, the higher binding capacity and stronger affinity of Capto Adhere toward both monomers and aggregates resulted in a less effective separation. However, it should be noted that this behavior may be influenced by the feed composition, where 60% of the total 10% aggregate content was prepared by thermal aggregation.

MMC-Ag4 thus appears to be a very promising prototype multimodal anion exchanger with an optimal combination of pore size and ligand density. The potential of some multimodal adsorbents bearing BMEA or structurally similar ligands to effectively separate IgG monomers and aggregates is known. However, our study shows that the separation efficiency is strongly dependent on pore structure. The density of the agarose matrix has been shown to affect the protein mass transfer rate and kinetics of adsorption [14,53], but not the selectivity of aggregate and monomer binding. The dependence of selectivity on agarose matrix density may be associated with its effect on the pore accessibility of aggregates with different molecular weights, which could be further amplified by pore blocking and electrostatic shielding effects of ligand-bound aggregates [54,55].

## 4. Conclusions

This study demonstrates that the separation of IgG monomers from protein aggregates using multimodal chromatography is critically dependent on a fine balance between resin pore structure and ligand density. The preparation of MMC-Ag4 and MMC-Ag6 resins resulted in a significant increase in matrix rigidity and dry matter content (up to 23%), indicating substantial syneresis of the agarose gel during functionalization. This structural modification transformed soft agarose beads into more rigid mesoporous adsorbents suitable for high-resolution protein separations.

Inverse size-exclusion chromatography (ISEC) and subsequent modelling revealed that while both resins maintained structural integrity, MMC-Ag4 possessed a larger mean pore radius (19.1 nm) compared to MMC-Ag6 (15.7 nm). This 20–25% difference in pore size proved to be the determining structural factor for the selectivity within the experimental scope of this study. While the denser MMC-Ag6 matrix showed nonselective binding, the broader pore structure of MMC-Ag4 facilitated preferential aggregate recognition, achieving a selectivity factor of approximately 2 in batch experiments. Geometric considerations suggest that the narrower pores of MMC-Ag6 are more susceptible to immediate blockage by larger aggregates, thereby hindering effective separation.

Chromatographic studies further confirmed the enhanced performance of the MMC-Ag4 prototype. In comparison to the commercial benchmark Capto Adhere, which exhibited stronger binding and lower yields under the tested conditions, MMC-Ag4 provided high-purity monomer fractions (97–98%) with excellent yields of 80–90%. Furthermore, the purification performance of MMC-Ag4 was maintained across the tested range of NaCl concentrations, indicating salt tolerance under these conditions. Although the thermally aggregated polyclonal IgG model system provided a reproducible source of aggregates, validation with industrial mAb feedstocks containing aggregates generated by shear, freeze–thaw, or other process-relevant stresses would further strengthen the generalizability of these findings. Collectively, these findings suggest that, within the scope of this study, base matrix density and pore accessibility play an important role alongside ligand chemistry in the design of multimodal adsorbents for the selective removal of protein aggregates.

## Figures and Tables

**Table 1:** Dry mass fraction and specific pore volume of the native and functionalized resins.

Resin	Dry Mass Fraction, *w* [%]	Specific Pore Volume *, *v*_p_ [mL/g]
4% B Agarose Bead	5.14	18.5
6% B Agarose Bead	6.41	14.6
MMC-Ag4	23.4	3.27
MMC-Ag6	22.4	3.46

* Per mass of dry resin.

**Table 2:** Adsorption characteristics of multimodal resins determined in batch experiments aggregates at pH 6.0.

Resin	*q_a_* [mg/g]	*q_m_* [mg/g]	*S* [−]
MMC-Ag4	5.2 ± 0.7	40.5 ± 3.3	1.75 ± 0.30
MMC-Ag6	4.5 ± 0.8	40.9 ± 4.0	0.99 ± 0.16

## Data Availability

The original data presented in this study are included in the article. Further inquiries can be directed to the corresponding author.

## Abbreviations

AEX	Anion exchange chromatography
BMEA	N-benzyl-N-methylethanolamine
CEX	Cation exchange chromatography
CHO	Chinese hamster ovary
DNA	Deoxyribonucleic acid
HCPs	Host cell proteins
HIC	Hydrophobic interaction chromatography
IgG	Immunoglobulin G
ISEC	Inverse size-exclusion chromatography
mAb	Monoclonal antibody
MMC	Mixed-mode chromatography
VEGF	Vascular endothelial growth factor
*A_evp_*	Integrated conductivity peak area [mL.mS/cm]
BCA	Bicinchoninic acid assay
*c_a_*	Equilibrium concentration of IgG aggregates [g/L]
*c_m_*	Equilibrium concentration of IgG monomers [g/L]
CV	Column volume [mL]
*q_a_*	Equilibrium adsorbed amount of IgG aggregates [mg/g]
*q_m_*	Equilibrium adsorbed amount of IgG monomers [mg/g]
*K_p_*	Distribution coefficient [-]
Λ*_max_*	Measured conductivity of 10 mM HCl solution [mS/cm]
Λ*_set_*	Theoretical conductivity of 10 mM HCl solution [mS/cm]
*m_M_*	Mass of the resin [g]
*M_w_*	Weight-average molecular weight [g/moL]
*r_h_*	Hydrodynamic radius [nm]
*r_p_*	Pore radius [nm]
*S*	Selectivity coefficient [−]
*t_i_*	Retention time of solute probe [min]
*t_max_*	Retention time of the largest dextran [min]
*t_min_*	Retention time of glucose [min]
*t_R_*	Retention time [min]
*v_p_*	Specific pore volume [mL/g]
*V_HCl_*	Volume of HCL solution [mL]
*V_tot_*	Dead volume of the system [mL]
*w*	Dry mass fraction [%]
*Λ*	Ligand density [µmol/g]

## References

[1] Joubert M.K., Hokom M., Eakin C., Zhou L., Deshpande M., Baker M.P., Goletz T.J., Kerwin B.A., Chirmule N., Narhi L.O. (2012). Highly Aggregated Antibody Therapeutics Can Enhance the in Vitro Innate and Late-stage T-cell Immune Responses. J. Biol. Chem..

[2] Wang W., Roberts C.J. (2010). Aggregation of Therapeutic Proteins.

[3] Roberts C.J. (2014). Therapeutic protein aggregation: Mechanisms, design, and control. Trends Biotechnol..

[4] Wang W. (2005). Protein aggregation and its inhibition in biopharmaceutics. Int. J. Pharm..

[5] Roberts C.J. (2014). Protein aggregation and its impact on product quality. Curr. Opin. Biotechnol..

[6] Wang W., Nema S., Teagarden D. (2010). Protein aggregation—Pathways and influencing factors. Int. J. Pharm..

[7] Weids A.J., Ibstedt S., Tamás M.J., Grant C.M. (2016). Distinct stress conditions result in aggregation of proteins with similar properties. Sci. Rep..

[8] Vázquez-Rey M., Lang D.A. (2011). Aggregates in monoclonal antibody manufacturing processes. Biotechnol. Bioeng..

[9] Housmans J.A.J., Wu G., Schymkowitz J., Rousseau F. (2023). A guide to studying protein aggregation. FEBS J..

[10] DiLeo M., Ley A., Nixon A.E., Chen J. (2017). Choices of capture chromatography technology in antibody manufacturing processes. J. Chromatogr. B.

[11] Farid S.S. (2008). Economic drivers and trade-offs in antibody purification processes. BioPharm. Int..

[12] Brenac Brochier V., Schapman A., Santambien P., Britsch L. (2008). Fast purification process optimization using mixed-mode chromatography sorbents in pre-packed mini-columns. J. Chromatogr. A.

[13] Aoyama S., Matsumoto Y., Mori C., Sota K. (2022). Application of novel mixed mode chromatography (MMC) resins having a hydrophobic modified polyallylamine ligand for monoclonal antibody purification. J. Chromatogr. B Anal. Technol. Biomed. Life Sci..

[14] Gao D., Wang L.L., Lin D.Q., Yao S.J. (2013). Evaluating antibody monomer separation from associated aggregates using mixed-mode chromatography. J. Chromatogr. A.

[15] Arora I. (2013). Chromatographic Methods for the Purification of Monoclonal Antibodies and their Alternatives A Review. Int. J. Emerg. Technol. Adv. Eng..

[16] Shukla A.A., Hubbard B., Tressel T., Guhan S., Low D. (2007). Downstream processing of monoclonal antibodies--application of platform approaches. J. Chromatogr. B Anal. Technol. Biomed. Life Sci..

[17] Fahrner R.L., Knudsen H.L., Basey C.D., Galan W., Feuerhelm D., Vanderlaan M., Blank G.S. (2001). Industrial purification of pharmaceutical antibodies: Development, operation, and validation of chromatography processes. Biotechnol. Genet. Eng. Rev..

[18] Chen J., Tetrault J., Ley A. (2008). Comparison of standard and new generation hydrophobic interaction chromatography resins in the monoclonal antibody purification process. J. Chromatogr. A.

[19] Ghose S., Tao Y., Conley L., Cecchini D. (2013). Purification of monoclonal antibodies by hydrophobic interaction chromatography under no-salt conditions. MAbs.

[20] Zhang K., Liu X. (2016). Mixed-mode chromatography in pharmaceutical and biopharmaceutical applications. J. Pharm. Biomed. Anal..

[21] Pinto I.F., Aires-Barros M.R., Azevedo A.M. (2015). Multimodal chromatography: Debottlenecking the downstream processing of monoclonal antibodies. Pharm. Bioprocess..

[22] Wei B., Dai L., Zhang K. (2025). Applications of hydrophilic interaction and mixed-mode liquid chromatography in pharmaceutical analysis. J. Chromatogr. A.

[23] Halan V., Maity S., Bhambure R., Rathore A.S. (2019). Multimodal Chromatography for Purification of Biotherapeutics—A Review. Curr. Protein Pept. Sci..

[24] Brekkan E., Jagschies G., Lindskog E., Łącki K., Galliher P. (2018). Chapter 20—Multimodal Chromatography. Biopharmaceutical Processing.

[25] Zhao G., Dong X.Y., Sun Y. (2009). Ligands for mixed-mode protein chromatography: Principles, characteristics and design. J. Biotechnol..

[26] Rupčíková V., Molnár T., Kurák T., Polakovič M. (2025). Antibody Aggregate Removal by Multimodal Chromatography. Molecules.

[27] Kaleas K.A., Tripodi M., Revelli S., Sharma V., Pizarro S.A. (2014). Evaluation of a multimodal resin for selective capture of CHO-derived monoclonal antibodies directly from harvested cell culture fluid. J. Chromatogr. B Anal. Technol. Biomed. Life Sci..

[28] Pizarro S.A., Gunson J., Field M.J., Dinges R., Khoo S., Dalal M., Lee M., Kaleas K.A., Moiseff K., Garnick S. (2010). High-yield expression of human vascular endothelial growth factor VEGF(165) in *Escherichia coli* and purification for therapeutic applications. Protein Expr. Purif..

[29] Kaleas K.A., Schmelzer C.H., Pizarro S.A. (2010). Industrial case study: Evaluation of a mixed-mode resin for selective capture of a human growth factor recombinantly expressed in E. coli. J. Chromatogr. A.

[30] Matos T., Queiroz J.A., Bülow L. (2014). Plasmid DNA purification using a multimodal chromatography resin. J. Mol. Recognit..

[31] Nfor B.K., Noverraz M., Chilamkurthi S., Verhaert P.D., van der Wielen L.A., Ottens M. (2010). High-throughput isotherm determination and thermodynamic modeling of protein adsorption on mixed mode adsorbents. J. Chromatogr. A.

[32] Cytiva (2020). Multimodal Chromatography: Handbook.

[33] Zhang X., Chen T., Li Y. (2019). A parallel demonstration of different resins’ antibody aggregate removing capability by a case study. Protein Expr. Purif..

[34] Pezzini J., Joucla G., Gantier R., Toueille M., Lomenech A.M., Le Sénéchal C., Garbay B., Santarelli X., Cabanne C. (2011). Antibody capture by mixed-mode chromatography: A comprehensive study from determination of optimal purification conditions to identification of contaminating host cell proteins. J. Chromatogr. A.

[35] Chen J., Tetrault J., Zhang Y., Wasserman A., Conley G., Dileo M., Haimes E., Nixon A.E., Ley A. (2010). The distinctive separation attributes of mixed-mode resins and their application in monoclonal antibody downstream purification process. J. Chromatogr. A.

[36] Maria S., Joucla G., Garbay B., Dieryck W., Lomenech A.-M., Santarelli X., Cabanne C. (2015). Purification process of recombinant monoclonal antibodies with mixed mode chromatography. J. Chromatogr. A.

[37] Toueille M., Uzel A., Depoisier J.-F., Gantier R. (2011). Designing new monoclonal antibody purification processes using mixed-mode chromatography sorbents. J. Chromatogr. B.

[38] Burton S.C., Harding D.R.K. (1997). Bifunctional etherification of a bead cellulose for ligand attachment with allyl bromide and allyl glycidyl ether. J. Chromatogr. A.

[39] Burton S.C., Harding D.R.K. (1997). High-density ligand attachment to brominated allyl matrices and application to mixed mode chromatography of chymosin. J. Chromatogr. A.

[40] Gao D., Yao S.J., Lin D.Q. (2008). Preparation and adsorption behavior of a cellulose-based, mixed-mode adsorbent with a benzylamine ligand for expanded bed applications. J. Appl. Polym. Sci..

[41] Lu H.-L., Lin D.-Q., Gao D., Yao S.-J. (2013). Evaluation of immunoglobulin adsorption on the hydrophobic charge-induction resins with different ligand densities and pore sizes. J. Chromatogr. A.

[42] Hagemann F., Adametz P., Wessling M., Thom V. (2020). Modeling hindered diffusion of antibodies in agarose beads considering pore size reduction due to adsorption. J. Chromatogr. A.

[43] Bernardi S., Gétaz D., Forrer N., Morbidelli M. (2013). Modeling of mixed-mode chromatography of peptides. J. Chromatogr. A.

[44] Molnár T., Rupčíková V., Kotuličová S., Polakovič M. (2025). A fluorescent dye assay for antibody aggregates and its application in aggregate stability evaluation. Acta Chim. Slovaca.

[45] Tatárová I., Gramblička M., Antošová M., Polakovič M. (2008). Characterization of pore structure of chromatographic adsorbents employed in separation of monoclonal antibodies using size-exclusion techniques. J. Chromatogr. A.

[46] Kurák T., Polakovič M. (2022). Adsorption Performance of a Multimodal Anion-Exchange Chromatography Membrane: Effect of Liquid Phase Composition and Separation Mode. Membranes.

[47] Sigma-Aldrich (2024). Bicinchoninic Acid Protein Assay Kit: Technical Bulletin.

[48] Grznárová G., Yu S., Štefuca V., Polakovič M. (2005). Quantitative characterization of pore structure of cellulose gels with or without bound protein ligand. J. Chromatogr. A.

[49] Rouquerol J., Avnir D., Fairbridge C., Everett D.H., Haynes J.H., Pernicone N., Ramsay J.D.F., Sing K.S.W., Unger K.K. (1994). Recommendations for the characterization of porous solids (Technical Report). Pure Appl. Chem..

[50] Barrande M., Beurroies I., Denoyel R., Tatárová I., Gramblička M., Polakovič M., Joehnck M., Schulte M. (2009). Characterisation of porous materials for bioseparation. J. Chromatogr. A.

[51] Franke A., Forrer N., Butté A., Cvijetić B., Morbidelli M., Jöhnck M., Schulte M. (2010). Role of the ligand density in cation exchange materials for the purification of proteins. J. Chromatogr. A.

[52] Wrzosek K., Polakovič M. (2011). Effect of pH on protein adsorption capacity of strong cation exchangers with grafted layer. J. Chromatogr. A.

[53] Fan J., Lavoie J., LeBarre J., Menegatti S., Pourdeyhimi B., Boi C., Carbonell R.G. (2025). High-capacity nonwoven increases productivity of mAb purification in an all-membrane process. Sep. Purif. Technol..

[54] Wrzosek K., Gramblička M., Polakovič M. (2009). Influence of ligand density on antibody binding capacity of cation-exchange adsorbents. J. Chromatogr. A.

[55] Forrer N., Kartachova O., Butté A., Morbidelli M. (2008). Investigation of the Porosity Variation during Chromatographic Experiments. Ind. Eng. Chem. Res..

